# Brief scales to assess physical activity and sedentary equipment in the home

**DOI:** 10.1186/1479-5868-7-10

**Published:** 2010-01-31

**Authors:** Dori E Rosenberg, James F Sallis, Jacqueline Kerr, Jason Maher, Gregory J Norman, Nefertiti Durant, Sion K Harris, Brian E Saelens

**Affiliations:** 1 Joint Doctoral Program in Clinical Psychology, San Diego State University and the University of California, San Diego, CA, USA; 2 Psychology Department, San Diego State University, San Diego, CA, USA; 3 Department of Family and Preventive Medicine, University of California, San Diego, La Jolla, CA, USA; 4 School of Medicine, University of Alabama at Birmingham, Birmingham, AL, USA; 5 Pediatrics, Children's Hospital Boston, Boston, MA, USA; 6 University of Washington and Seattle Children's Hospital Research Institute, Seattle, WA, USA

## Abstract

**Background:**

Sedentary behaviors such as TV viewing are associated with childhood obesity, while physical activity promotes healthy weight. The role of the home environment in shaping these behaviors among youth is poorly understood. The study purpose was to examine the reliability of brief parental proxy-report and adolescent self-report measures of electronic equipment and physical activity equipment in the home and to assess the construct validity of these scales by examining their relationship to physical activity, sedentary behavior, and weight status of children and adolescents.

**Methods:**

Participants were adolescents (n = 189; mean age = 14.6), parents of adolescents (n = 171; mean age = 45.0), and parents of younger children (n = 116; parents mean age = 39.6; children's mean age = 8.3) who completed two surveys approximately one month apart. Measures included a 21-item electronic equipment scale (to assess sedentary behavior facilitators in the home, in the child or adolescent's bedroom, and portable electronics) and a 14-item home physical activity equipment scale. Home environment factors were examined as correlates of children's and adolescents' physical activity, sedentary behavior, and weight status after adjusting for child age, sex, race/ethnicity, household income, and number of children in the home.

**Results:**

Most scales had acceptable test-retest reliability (intraclass correlations were .54 - .92). Parent and adolescent reports were correlated. Electronic equipment in adolescents' bedrooms was positively related to sedentary behavior. Activity equipment in the home was inversely associated with television time in adolescents and children, and positively correlated with adolescents' physical activity. Children's BMI z-score was positively associated with having a television in their bedroom.

**Conclusions:**

The measures of home electronic equipment and activity equipment were similarly reliable when reported by parents and by adolescents. Home environment attributes were related to multiple obesity-related behaviors and to child weight status, supporting the construct validity of these scales.

## Background

Sedentary behaviors require low energy expenditure and include time spent sitting while watching television (TV) and movies, playing video games, and using the computer [1,2]. According to the Centers for Disease Control and Prevention (CDC), more than one third of U.S. adolescents watch more than 3 hours per day of TV [2], despite recommendations to limit exposure to less than 2 hours per day [3]. Sedentary behaviors, especially TV watching, are among the most consistent behavioral correlates of obesity in youth [1]. Interventions to decrease time spent watching TV and using computers have helped to reduce BMI in children [4-6].

Low levels of physical activity contribute to youth obesity independently of sedentary behaviors [7-9]. Recent data with objective monitoring in the U.S. indicated that children between ages 6 and 11 spent about 6 hours per day engaged in sedentary behavior (<100 accelerometer counts/minute), with 7.5 hours for those aged 12-15 and over 8 hours for those ages 16-19 [10]. Objectively measured data from the European Youth Heart Study indicated that the percent of time spent being sedentary (<500 accelerometer counts/minute) was 62.2 for 9 year old boys, 66.3 for 9 year old girls, 71.5 for 15 year old boys, and 75.8 for 15 year old girls [11].

Along with individual and social environment influences from family and friends, obesogenic environmental attributes of homes, neighborhoods, schools, and elsewhere are believed to promote physical inactivity and sedentary behaviors among youth [12]. Among children and adolescents, having a TV in the bedroom was associated with more TV time [13-17], less physical activity [16,17], poor eating habits [16], more likelihood of being overweight [15,17,18], and poor school performance [16]. Having access to more TVs in the home has been associated with more time watching TV [13,19]. Some studies have found that after adjustment for other family factors, TVs in the bedroom were not related to more TV time [20]. Exercise equipment in the home may be positively related to physical activity, particularly in adolescent girls [21], but the limited number of studies to date have not shown a relationship between exercise equipment in the home and youth obesity or sedentary behaviors [22,23].

Ecological models are commonly used to guide studies of physical activity and sedentary behavior, and they posit multiple levels of influence ranging from individual to environmental and policy factors [24,25]. There are likely multiple pathways by which environmental factors, including electronic and exercise equipment in the home, can affect behaviors, such as providing opportunities, providing cues, signaling parental values and support, and through effects on parent or sibling behavior. Young people are exposed to their home environments daily over many years. Thus, it is important to identify whether and which attributes of the home environment are related to physical activity and sedentary behaviors, and therefore likely affect weight status. Simple, reliable, and valid measures of the home environment are needed to conduct high quality research on potential pathways by which home environment attributes may affect physical activity and sedentary behaviors.

Of the measures used to assess home environments, some have been evaluated for reliability [6,13,26,27] and others have not [23]. Recently published measures of sedentary equipment [26,27] are lengthy (30-45 minutes to complete) and intended for completion by parents, not youth, and have not been studied in relation to behaviors and health outcomes. A previous measure of home activity equipment was designed for adults and has not been adapted or evaluated for youth [28]. The present study evaluated the reliability and construct validity of new, brief measures of the home environment for sedentary and physical activity equipment. Parallel versions were developed for parents and adolescents to complete, allowing researchers more flexibility for use. As part of the validity analysis for this investigation, the relationships between the home environment measures and youth physical activity, sedentary behavior, and weight status were explored.

## Methods

Participants were part of a larger study examining the measurement of the neighborhood environment in relation to a variety of outcomes including eating behavior, physical activity and sedentary behaviors. Three groups of participants were recruited. The first group was parents of children (n = 116; children ages 5 to 11) who were on average 39.6 years old (SD = 7.7), 77.6% white, and 86.1% female. The second group was parents of adolescents (n = 171; adolescents ages 12 to 18) who had a mean age of 45.0 years (SD = 6.8), were 57.3% white, and were 80.5% female. The third group was 189 adolescents between the ages of 12 and 18. The parents of adolescents were paired with their corresponding adolescent (N = 171 pairs) for construct validity analyses. Participants were recruited from three United States cities: San Diego, CA, Boston, MA and Cincinnati, OH.

### Recruitment

The goal was to recruit a sample diverse in neighborhood and sociodemographic characteristics since the study design required a range of neighborhood environment attributes. However, for the current investigation, neighborhood environment was not a variable of interest. More specific recruitment details have been published previously [29,30]. Recruitment methods varied in each geographic location and included mail and telephone contact of potential participants identified by a commercial marketing firm and in-person contact through schools, community events, and recreation facilities. The study was described as investigating neighborhood characteristics, physical activity, and nutrition. Survey 1 was given to or mailed to participants with a pre-paid return envelope. Approximately 10 days after return receipt of survey 1, survey 2 was sent to participants with a pre-paid return envelope. Average time to complete the entire survey was 30-45 minutes while average time to complete the home environment and physical activity equipment measures was 5-10 minutes. Participants received a $20 incentive after completion of both questionnaires. Ethical approval was obtained from the respective institutions in each city. Informed consent/assent was obtained from participants.

### Procedure

A test-retest design was utilized. Participants were asked to note "today's date" at the end of the survey. The average time between completion of the two surveys based on response to this item was 27 days. The response rate varied by study site and recruitment method. San Diego used a combination of "cold-calling" and in-person recruitment at local community centers and events. Of those called, 54% of those eligible who agreed to participate by phone completed at least one survey. Of those recruited at community centers and events, 15% of those given surveys completed at least one survey. For the Cincinnati site, families received a study introductory letter and at least one telephone call to assess their interest and eligibility. Of those who agreed to participate, 73% returned at least one survey. In Boston, the agreed-to-participate rate following face-to-face contact and phone prompting was 47.5%. Across sites, 74% percent of parents and 62% of adolescents who consented to participate completed both survey 1 and 2. For the current reliability analyses, all three participant groups, parents of children, parents of adolescents, and adolescents completed survey 1 and 2, while those completing at least survey 1 were included in cross-sectional analyses.

### Item Development

The survey was developed from existing measures [31], previous research [13,28,32,33], and through a formative research process. This formative process included phone and in-person interviews conducted with children and parents in San Diego and Cincinnati. Participants in the formative process did not take part in the larger study.

### Measures

All survey measures were completed by all 3 participant groups: parents as a proxy for their children between ages 5 and 11, parents as a proxy for their adolescents between ages 12 and 18, and adolescents for themselves. Wording was slightly different on the measure depending on whether the respondent was answering as a proxy for their child or adolescent or for themselves (in the case of adolescents).

Two brief measures of home environments, taking approximately 5-10 minutes to complete, were evaluated in the current analyses. For the first measure, termed the home electronic equipment scale, participants recorded the number of various types of electronic entertainment or information devices available in the home and in the child/adolescent's bedroom using an open-ended format. There were 3 subscales: electronics available in the home (8 items), electronics available in the child's or adolescent's bedroom (8 items), and portable electronics (5 items). See Table 1 for the types of electronics evaluated. The number of items was summed for each subscale to create the total count of electronics in the home (not excluding those available in the youth's bedroom), electronics in the youth's bedroom, and portable electronics. The number of TVs in the home and presence of a TV in the child or adolescent's bedroom were examined as part of the subscales and also separately.

**Table 1:** Descriptive statistics for electronic equipment in the home and bedroom and portable electronic equipment

	Mean (SD, range) in home	% with at least 1 in the home	Mean (SD, range) in bedroom	% with at least 1 in the bedroom	Portable electronics mean (SD & range)	% with at least 1
Adolescent reported						
TVs	3.7 (1.6, 0-8)	99.4	.6 (.5, 0-3)	53.8	n/a	n/a
VCR/DVD	3.2 (1.8, 0-10)	98.7	.5 (.7, 0-5)	43.9	n/a	n/a
Digital TV recorders	.5 (.9, 0-5)	29.1	.07 (.3, 0-3)	5.7	n/a	n/a
Music players	4.0 (2.0, 0-10)	96.9	1.3 (.9, 0-6)	94.9	2.5 (1.7, 0-10)	94.3
Desktop computer with internet	1.6 (1.2, 0-8)	90.0	.2 (.4, 0-2)	23.5	n/a	n/a
Desktop computer without internet	.41 (.8, 0-6)	31	.2 (.5, 0-4)	11.5	n/a	n/a
Video game player	1.3 (1.2, 0-6)	71.2	.4 (.7, 0-4)	25.8	1.2 (1.3, 0-6)	62.5
Telephone (non cell phone)	3.5 (1.8, 0-10)	96.9	.5 (.5, 0-2)	43.9	n/a	n/a
Laptop with internet	n/a	n/a	n/a	n/a	.9 (1.0, 0-5)	57.5
Laptop without internet	n/a	n/a	n/a	n/a	.3 (.7, 0-4)	25.3
Cell phones	n/a	n/a	n/a	n/a	2.5 (1.5, 0-7)	89.9
Total subscale	17.6 (6.1, 4-39)	n/a	3.5 (2.2, 0-11)	n/a	7.3 (3.5, 0-19)	n/a
						
Adolescents (parent reported)						
TVs	3.6 (1.6, 0-8)	99.4	.6 (.6, 0-3)	52.9	n/a	n/a
VCR/DVD	2.9 (1.7, 0-11)	98.2	.4 (.6, 0-2)	39.0	n/a	n/a
Digital TV recorders	.5 (1.1, 0-7)	29.4	.1 (.3, 0-2)	8.9	n/a	n/a
Music players	3.7 (1.7, 0-9)	98.2	1.1 (.7, 0-5)	82.7	2.2 (1.4, 0-6)	94.7
Desktop computer with internet	1.5 (1.1, 0-6)	91.2	.2 (.4, 0-2)	22.9	n/a	n/a
Desktop computer without internet	.3 (.6, 0-3)	26.0	.1 (.3, 0-2)	8.5	n/a	n/a
Video game player	1.2 (1.1, 0-7)	70.2	.4 (.7, 0-4)	27.5	1.1 (1.3, 0-8)	63.2
Telephone (non cell phone)	3.3 (1.7, 0-10)	98.8	.4 (.5, 0-2)	39.2	n/a	n/a
Laptop with internet	n/a	n/a	n/a	n/a	.9 (1.1, 0-6)	58.2
Laptop without internet	n/a	n/a	n/a	n/a	.3 (.6, 0-3)	23.1
Cell phones	n/a	n/a	n/a	n/a	2.0 (1.4, 0-7)	86.5
Total subscale	16.5 (5.6, 5-43)	n/a	3.1 (2.1, 0-11)	n/a	6.5 (3.5, 0-23)	n/a
						
Children (parent reported)						
TVs	2.8 (1.2, 0-7)	99.1	.4 (.5, 0-2)	39.5	n/a	n/a
VCR/DVD	2.4 (1.3, 0-6)	99.1	.3 (.5, 0-2)	29.4	n/a	n/a
Digital TV recorders	.3 (.7, 0-3)	19.8	.1 (.3, 0-2)	4.6	n/a	n/a
Music players	2.9 (1.4, 1-7)	81.6	.8 (.7, 0-3)	67.0	1.3 (1.3, 0-6)	70.7
Desktop computer with internet	1.1 (.8, 0-4)	81.0	.1 (.3, 0-1)	9.2	n/a	n/a
Desktop computer without internet	.2 (.5, 0-2)	23.4	.04 (.2, 0-1)	3.8	n/a	n/a
Video game player	.9 (1.1, 0-5)	59.6	.2 (.5, 0-3)	17.3	.8 (1.0, 0-5)	52.2
Telephone (non cell phone)	2.6 (1.4, 0-9)	98.2	.1 (.3, 0-1)	13.1	n/a	n/a
Laptop with internet	n/a	n/a	n/a	n/a	.6 (.7, 0-3)	53.9
Laptop without internet	n/a	n/a	n/a	n/a	.1 (.4, 0-2)	13.4
Cell phones	n/a	n/a	n/a	n/a	.8 (1.0, 0-3)	43.9
Total subscale	13.1 (4.6, 4-28)	n/a	1.9 (1.7, 0-7)	n/a	3.6 (2.9, 0-13)	n/a

The second measure was termed the "home physical activity equipment scale." The scale was adapted from an earlier version developed for adults [28] based on the formative research phase. The measure consisted of a checklist (yes/no response) of the availability of 14 types of physical activity equipment and supplies in or around the home (bikes, basketball hoop, jump rope, sports equipment, swimming pool, roller skates, fixed play equipment, home aerobic equipment, weight lifting equipment, water or snow equipment, yoga/exercise mats, exercise/play/recreation room, trampoline, and stairs). The score was a tally of available equipment or supplies.

All three participant groups (parents as a proxy for their child, parents as a proxy for their adolescent, and adolescents) completed four outcome measures (TV watching, sedentary behavior, physical activity, and BMI) that were used for construct validity analyses.

Child and adolescent sedentary behavior was measured in time per typical week spent on various sedentary behaviors (watching TV, using a computer, driving in a car, playing video games, sitting and listening to music, sitting and talking on the phone, sitting to hang out with others, reading a book, doing inactive hobbies), adapted from previous measures [6,22]. The questions were asked for a usual weekday and weekend day separately. Response options were: none, 15 minutes, 30 minutes, 1 hour, 2 hours, 3 hours, or 4 or more hours. Responses were recoded into duration of time spent on each sedentary behavior (e.g. 15 minutes recoded as .25 hours; 4 or more hours recoded as 4.0 hours), summed across all items, and multiplied by the appropriate days per week (5 for weekday and 2 for weekend day). The final score, sedentary composite hours per week, was the sum of weekday and weekend sedentary time. Hours/week spent watching TV was used as a separate, additional outcome.

Youth moderate-to-vigorous physical activity was assessed with a two-item scale. Survey instructions defined moderate-to-vigorous physical activity as producing an increase in heart rate and breathing "some of the time", with several examples. The first item was, "Over the past 7 days, on how many days were you physically active for a total of at least 60 minutes per day?" The second item was similar in wording, but it asked about physical activity during a "typical or usual week". Mean days in the past week and typical week were averaged. In prior studies, this measure correlated significantly with accelerometer data (r = .40), and was supported by sensitivity/specificity analyses [34]. Physical activity measured this way has been associated with lower odds of being overweight in 29 of 33 countries in an international study in youth [35]. Although the measure has only been validated as an adolescent self-report, in the present study it was modified for parents to report on their children's and adolescents' physical activity.

Body mass index (BMI) was based on self-reported height and weight by adolescents and parent-report of their child or adolescent's height and weight. BMI was calculated as: weight (kg)/height (m)^2^. BMI z-score was determined from CDC national norms using age to the nearest month and sex-specific median, standard deviation, and power of the Box-Cox transformation [36]. While it is not ideal to use self- and parent-reported height and weight, it was not feasible to use an objective measurement as the study was conducted primarily through mailed surveys. Studies have found reasonable intraclass correlations for adolescents' self-reports of height (range from .57-.91) and weight (range from .85-.98) [37]. However, several studies have demonstrated that adolescents tend to over-report height and under-report weight [38-40]. Some studies have suggested that the under-reporting does not largely affect obesity classification. For example, one study among 12-16 year olds, showed that weight status was correctly classified for 94% of the sample [41].

Adolescents self-reported their gender and age. Parents also reported their child's gender and age. Adolescents reported their race/ethnicity, while parent race/ethnicity was used as a proxy for child race/ethnicity. Only parents reported the household income and number of children (under age 18) living in the household.

### Analysis

One-way random effects models for intra-class correlation coefficients (ICCs) were calculated to assess test-retest reliability of equipment counts for each item in the home environment measures. Reliability was then evaluated for each equipment scale (home, bedroom, portable), separately for adolescents (12-18 years), parents of adolescents (12-18 years) and parents of children (5-11 years). Adolescent-parent agreement ICCs were calculated at time 1 only. The ICCs were interpreted following benchmarks of <.10 virtually none, .11 to .40 slight, .41 to .60 fair, .61 to .80 moderate, .81 to 1.0 substantial [42]. Thus, ICCs > .60 were considered acceptable.

Construct validity was assessed using hierarchical linear regression models. In block 1, demographic variables, which included gender (male or female), child age, race/ethnicity (white or non-white), household income (less than $50,000 per year or $50,000+), and number of children in the household were entered. In block 2, each subscale (electronic equipment in the home, number of TVs in the home, electronic equipment in the bedroom, portable electronic equipment, or home activity equipment) was entered in separate models. The dependent variables were time spent watching TV, sedentary composite score (including TV watching), physical activity, and BMI z-score; each was examined in a separate model. The reporter (parents as a proxy for their child, parents as a proxy for their adolescent, and adolescents) was kept consistent in models (e.g. if the outcome was adolescent reported physical activity, all adolescent reported variables were used in models). The only exception was for household income and number of children in the household which were reported only by parents. Additionally, child race/ethnicity was not reported by parents so parent race/ethnicity was used as a proxy in models where parents reported for their child.

Analysis of Covariance was used to assess differences in the four outcomes based on whether a child or adolescent did or did not have a TV in his/her bedroom. For all analyses, except adolescent reported reliability, only data from matching parent-adolescent pairs were used (N = 171 pairs).

## Results

The children for whom parents reported were on average 8.3 years of age (SD = 1.9), 52.2% were female, and 32% were above the 85^th ^percentile for age and sex specific BMI. Adolescents were on average 14.6 years of age (SD = 1.7), 50.7% female, 62% white, and 32% were above the 85^th ^percentile for height and weight based on adolescent reports (32.7% based on parent report). For children, 58.6% of the sample had household income above $50,000/year while 61.0% of adolescent households had income above $50,000/year.

Table 1 presents the means, standard deviations, and ranges for each of the electronic equipment items and subscales. The table also shows the percent of participants who had at least one of each item. Nearly 54% of adolescents had a TV in their bedroom while about 39% of children did. The most common types of home activity equipment available for both adolescents (self-reported) and children (parent reported) were: sports equipment (82% of adolescents; 92% of children), bikes (80% of adolescents, 90% of children), stairs (80% of adolescents and children), jump ropes (59% of adolescents, 79% of children), and skateboards/scooters/roller skates (70% of adolescents, 85% of children). Table 2 presents the means and standard deviations for the outcome variables.

**Table 2:** Descriptive data for outcome variables

	Parental Proxy-report for child (N = 116)		Parental Proxy-report for adolescent (N = 171)		Self-report by adolescent (N = 189)	
	Mean	SD	Mean	SD	Mean	SD
TV viewing (hours/week)	24.9	9.3	26.4	10.4	26.3	11.1
Sedentary composite (hours/week)	115.4	41.2	155.1	51.5	166.6	59.2
Physical activity (days/week)	4.7	1.8	3.7	2.2	4.0	2.2
BMI	18.8	5.5	22.3	5.2	21.9	4.7

### Test-retest Reliability

The majority of ICCs for items were in acceptable ranges (i.e., >.60) for at least one responder group (see Table 3). While portable computers without the internet (ICC range = .37-.58) and swimming pools (ICC range .53-.58) were below the .60 cutoff, these two items were kept because they were very close to being within range. Though these marginal items were retained for present analyses, they could be deleted in future uses of the scale. Digital TV recorders had consistently low ICCs (.26 to .39); thus, this item was removed from the home electronic equipment scale. Test-retest reliabilities for the scales ranged from an ICC of .54 for home activity equipment (based on parent of adolescent reports) to an ICC of .92 for home electronics (based on parent reports for their children) (see Table 3). While parent-adolescent agreement was good (see Table 3), adolescents had consistently higher test-retest reliability for all equipment scales than parents. ICCs for the outcome variables were also in the acceptable range (see Table 3). The ICC between parent and adolescent reports of adolescent BMI was excellent (ICC = .87).

**Table 3:** Intra-class correlation coefficients for all scales and outcomes by each reporting group

Item	Parental Proxy-report for child (N = 116)	Parental Proxy-report for adolescent (N = 171)	Self-report by adolescent (N = 189)	Parent-adolescent agreement* (N = 171)
**Electronics in the home and bedroom**				
TVs	.96	.91	.87	.93
VCR/DVD player	.83	.60	.81	.75
Digital TV recorder	.26	.55	.39	.36
Music Player	.55	.38	.58	.64
Desktop computer with internet	.75	.76	.59	.72
Desktop computer without internet	.61	.59	.51	.50
Video game player	.87	.83	.78	.83
Telephone	.91	.82	.84	.80
Electronics in the Home Scale	.92	.71	.87	.82
Electronics in the Bedroom Scale	.90	.67	.88	.74
**Portable electronics**				
Music player	.59	.54	.38	.61
Handheld videogame player	.51	.63	.76	.74
Computer with internet access	.61	.59	.54	.59
Computer without internet	.37	.46	.58	.54
Cell phone	.67	.55	.64	.49
Portable Electronics Scale	.71	.56	.60	.63
**Physical activity equipment**				
Bikes	.85	.73	.78	.64
Basketball	.66	.76	.56	.70
Jump rope	.71	.72	.54	.45
Sports equip	.65	.50	.63	.44
Swimming pool	.53	.58	.48	.56
Roller skates	.74	.54	.51	.57
Fixed play equipment	.73	.50	.55	.49
Aerobic equip	.71	.70	.60	.68
Weight lifting	.58	.63	.59	.53
Water/snow equip	.67	.55	.62	.54
Yoga/exercise mat	.67	.54	.61	.52
Exercise, play, recreation room	.53	.66	.54	.51
Trampoline	.84	.62	.65	.59
Stairs	.84	.73	.54	.69
Physical Activity Equipment Scale	.80	.54	.69	.63
**Behavioral Outcomes**				
TV time	.67	.70	.63	.57
Sedentary Composite Score	.53	.62	.67	.54
Physical activity frequency	.65	.57	.63	.55

* Agreement calculated using time 1 data

### Construct Validity

Based on adolescent self-reports, TVs in the home was positively related to TV viewing time while activity equipment was inversely related to TV viewing. Electronic equipment in the bedroom and portable electronics were positively related to the sedentary behavior composite score (see Table 4). Home physical activity equipment was positively related to physical activity. Electronics in the bedroom was positively related to BMI z-score. Adolescents with a TV in their bedroom watched more TV and engaged in more sedentary behavior (see Table 5) than those without a TV in their bedroom.

**Table 4:** Construct validity analyses examining the associations between each subscale and the outcome variables

	Television viewing time			Sedentary composite			Physical activity			Body mass index z score		
	β	p	Δ R2	B	p	Δ R2	β	p	Δ R2	β	p	Δ R2
**Adolescent report**												
Electronics in home	.01	.93	.00	.13	.11	.02	.00	.99	.00	.15	.08	.02
TVs in the home	**.17**	**.03**	**.03**	.10	.19	.01	-.04	.59	.00	.08	.35	.01
Electronics in bedroom	.13	.12	.02	**.22**	**.005**	**.05**	.01	.92	.00	**.19**	**.03**	**.03**
Portable electronics	-.12	.13	.01	**.16**	**.047**	**.02**	.00	.98	.00	-.02	.84	.00
Activity equipment	**-.21**	**.01**	**.04**	.01	.91	.00	**.22**	**.01**	**.04**	-.10	.28	.01
**Parent-report of Adolescents**												
Electronics in home	.03	.72	.001	.09	.27	.01	.05	.53	.00	.12	.17	.01
TVs in the home	**.24**	**.00**	**.06**	**.19**	**.01**	**.04**	-.03	.71	.00	-.02	.98	.00
Electronics in bedroom	.07	.39	.005	.14	.07	.02	.10	.24	.01	**.17**	**.05**	**.03**
Portable electronics	**-.18**	**.03**	**.03**	.11	.18	.01	.06	.47	.00	.06	.53	.00
Activity equipment	**-.23**	**.003**	**.05**	-.07	.35	.005	**.20**	**.01**	**.04**	.05	.53	.00
**Parent-report of Children**												
Electronics in home	**.29**	**.006**	**.07**	.06	.54	.003	-.18	.09	.03	.09	.43	.01
TVs in the home	**.39**	**.00**	**.15**	.11	.24	.01	-.14	.15	.02	.10	.32	.01
Electronics in bedroom	.19	.11	.03	.12	.28	.01	-.04	.74	.00	.22	.08	.03
Portable electronics	.06	.58	.003	.13	.21	.01	-.07	.51	.004	.10	.36	.01
Activity equipment	**-.23**	**.02**	**.05**	-.16	.10	.02	.13	.18	.02	-.19	.07	.03

Note: Significant (p < .05) associations are in bold. Means adjusted for child age, gender, race/ethnicity, household income, and number of children living in the household.

**Table 5:** Outcomes comparing those with and without a TV in the bedroom

	No TV in Bedroom* (Mean)	TV in bedroom* (Mean)	**p-value**	**Partial Eta^2^**
	
**Adolescents**	**N = 73**	**N = 85**		
TV viewing (hours/week)	22.6	29.8	.00	.11
Sedentary composite (hours/week)	153.6	178.3	.01	.05
Physical activity (days/week)	4.2	3.8	.27	.01
BMI z-score	.31	.40	.65	.00
**Parents of adolescents**	N = 80	N = 90		
TV viewing (hours/week)	22.8	29.3	.00	.10
Sedentary composite (hours/week)	141.8	165.6	.01	.06
Physical activity (days/week)	3.6	3.8	.60	.00
BMI z-score	.41	.34	.77	.00
**Parents of children**	N = 69	N = 45		
TV viewing (hours/week)	22.7	27.7	.02	.06
Sedentary composite (hours/week)	114.4	117.2	.76	.00
Physical activity (days/week)	5.0	4.3	.11	.03
BMI z-score	.26	.94	.02	.06

Note: Means adjusted for child age, gender, race/ethnicity, household income, and number of children living in the household.

Slightly different relationships were observed when parent reports of their adolescent's behavior were considered (see Table 4). Number of TVs in the home was positively related to adolescents' TV viewing time while portable electronics and activity equipment were inversely related to TV viewing time (see Table 4). Number of TVs in the home was also positively related to the sedentary behavior composite score. Parent reports of physical activity equipment availability in the home were positively related to physical activity, as found with adolescent's self-report. There was a trend for electronics in the bedroom to be positively related to adolescent BMI z-score (p = .05). Also similar to adolescents' self-report, based on parent report, adolescents with a TV in their bedroom spent significantly more time watching TV and engaging in more sedentary behavior (see Table 5). Based on parent report, adolescents' BMI z-scores did not differ between those with versus without a TV in their bedroom.

Based on parent reports for younger children, electronic equipment in the home and number of TVs in the home were positively related to children's TV viewing (see Table 4). Home physical activity equipment was negatively associated with TV viewing. There were trends suggesting that electronics in the bedroom was positively associated with BMI z-score (p < .08) while physical activity equipment was negatively associated with BMI z-score (p < .07).

The amount of variance in the outcomes explained by home environment scales was small (range Δ R2 = .00 to .15). Children with a TV in their bedroom watched significantly more TV, but did not engage in more sedentary behavior than those without a TV in their bedroom. However, children with a bedroom TV had significantly higher BMI z-scores than children who did not (see Table 5).

## Discussion

The current study evaluated new brief measures of home environment factors hypothesized to be related to youth physical activity and sedentary behaviors. The measures demonstrated good evidence of test-retest reliability and moderate support for construct validity based on both parent and adolescent reports. The present measures appear to be the first to measure home environment constructs by adolescent report.

In addition to finding that the overall quantity of sedentary behavior facilitators (e.g., TVs, computers) were positively related to sedentary behavior for children and adolescents, having sedentary equipment in the bedroom appeared to be consistently related to higher TV viewing time and sedentary behavior, confirming previous studies. Children who had a TV in their bedroom had higher BMI z-scores than children who did not. Amount of electronic equipment in the bedroom was related to higher BMI z-scores for adolescents, significantly when adolescents self-reported and trends for parent report of their adolescents and children. Adolescents with a TV in their bedroom watched more TV and engaged in more sedentary behavior overall than those without a TV in their bedroom. Previous studies identified having a TV in the child's bedroom as related to high levels of TV viewing [13,14,16,43] and higher child weight status [15,17,18]. The results of the current study underscore the importance of discouraging TVs in the bedroom as an intervention target for child and adolescent obesity control.

At least one previous study found that accounting for other aspects of the family environment, such as parent TV watching and restricting TV during meals, could attenuate associations between youth TV availability and viewing, so further research is needed [20]. The present study adjusted for number of children in the home (as did Salmon et al., 2005; [20]), but still found significant associations. Perhaps the difference is related to the higher proportion of the U.S. children (40%) and adolescents (53%) in the current study having televisions in their bedrooms compared to the Salmon et al. 2005 [20] study in Australian 10-12 year olds (32.1% of boys and 24.6% of girls had TVs in their bedrooms) which adjusted for more family variables. Additionally, while parent rules may be an important factor accounting for youth time spent being sedentary, a home that has high access to TVs and many rules to limit TV time can send mixed messages to children. Rules may not be enough to limit TV time; a better approach may be to set rules and limit the availability of TVs in the home and child's bedroom.

This was the first study to test the reliability of reporting portable electronic equipment and relate it to behavior--an important addition to sedentary behavior research considering the growing trend for portable electronic equipment use by youth [44]. The finding that number of portable electronics was positively correlated with sedentary behavior among adolescents but negatively correlated with TV viewing time (based on parent reports) is notable and worthy of further investigation. This pattern of findings suggests that portable electronics could have differential effects on overall versus specific sedentary behaviors, so research is especially needed to explore the potential for positive effects of portable electronics. For example, portable electronics (e.g. portable music players, cell phones) could be used during physical activity and simultaneously substitute for time spent being sedentary while using other kinds of electronics.

There were consistent patterns for both children and adolescents that physical activity equipment in the home was inversely related to TV viewing; previous studies have not demonstrated this relationship. Also, home activity equipment was positively related to physical activity among adolescents, whether self or parent reported, supporting findings from previous research [21,22]. These findings support the construct validity of the home activity equipment measure and suggest the presence of such equipment could both facilitate physical activity and provide cues to reduce TV viewing. However, caution is warranted as our study is cross-sectional and further investigations using longitudinal and intervention research are needed to confirm these findings.

In addition to evidence of test-retest reliability and initial support for construct validity, there were other indicators of good psychometric performance of the new home environment survey measures. The surprising consistency of validity results across adolescent or parent reports and the moderate to strong correlations among adolescent and parent reports on the home environment measures indicate both versions provide similar data and may be useful as alternate versions. Although the pattern of findings was somewhat different for adolescents and children, significant associations with health-related outcomes in expected directions were found for both age groups. Two items (e.g. laptops without internet, swimming pools) had test-retest reliabilities that were slightly below criteria. In future studies, investigators may want to remove these items from the scales, unless it is desirable to retain the items for descriptive purposes. The present scales complement recently published longer home environment measures [26,27] and can be used in studies of youth physical activity, sedentary behaviors, and weight status.

Strengths of the study were development of new measures applicable to a wide age range of youth, parallel forms for parent and adolescent completion, recruitment from three regions of the United States, and examination of multiple construct validity outcomes. It was especially important to adjust for household income in the analyses since ability to purchase equipment could confound associations. However, future studies using structural equation modeling and accounting for other factors that may attenuate these associations (e.g. family rules, number of siblings) could advance understanding.

The primary limitation was reliance on self-reported behaviors and weight status for the construct validity measures. There is also the potential for method bias that could inflate associations among reported variables. Especially for young children, parent reports of child heights and weights are known to have limited reliability [45]. In the present study, agreement between parent and adolescent reports of adolescent BMI was excellent. Nevertheless, errors in self-reported BMI reduced power to detect associations as part of construct validity analyses. The average reported time spent in sedentary behaviors exceeded the amount possible in a day, suggesting that objective measures are needed. However, the sedentary behaviors were not mutually exclusive categories, and simultaneous use of multiple devices is possible (e.g. a child can watch TV while they are on the computer) [44]. The physical activity measure has not been validated for parent report of youth physical activity, though the test-retest reliability was acceptable in the present sample. An additional limitation is that it was not possible to adjust analyses for clustering within cities due to the small number of units and the study sample was not nationally representative. The study was cross-sectional so no causal interpretations can be made. We encourage future studies to examine moderators of home environment associations with youth outcomes, such as parent rules and behavior, physical activity opportunities in the neighborhood, and youth preferences.

## Conclusions

These new brief measures of home environment variables are ready for use in other studies to improve understanding of the many factors involved in the etiology of physical activity, sedentary behaviors, and obesity among children and adolescents, as well as to evaluate intervention effects (measures can be downloaded from http://www.drjamessallis.sdsu.edu or http://www.activelivingresearch.org). Future research directions include examining the home environment measures in relation to objectively measured outcomes, examining their performance in specific population subgroups, evaluating their ability to detect changes over time in home environments, and examining how the measures perform in the broader family environment. Electronics in the bedroom and home activity equipment had the strongest evidence of construct validity, and these are candidates for environmental changes that could be incorporated into intervention trials offered through schools, health care settings, or the mass media. The present study provides strong support for American Academy of Pediatrics recommendation for parents: "Remove television sets from children's bedrooms" [3].

## Competing interests

The authors declare that they have no competing interests.

## Authors' contributions

All authors have read and approved the final manuscript

DR helped design the study, drafted the manuscript, and performed statistical analysis

JS conceived the study, participated in the design of the study, and contributed to drafting and editing the manuscript

JK participated in the conception and design of the study and editing of the manuscript

JM assisted with drafting the manuscript and running analyses

ND participated in the design of the study and editing the manuscript

SKH participated in the design of the study and editing the manuscript

BES participated in the conception and design of the study and contributed to drafting and editing the manuscript
